# Phenotypic Characterization and Whole Genome Analysis of a Strong Biofilm-Forming *Staphylococcus aureus* Strain Associated With Subclinical Bovine Mastitis in Colombia

**DOI:** 10.3389/fvets.2020.00530

**Published:** 2020-09-04

**Authors:** Giovanny Torres, Karen Vargas, Yesid Cuesta-Astroz, Julián Reyes-Vélez, Martha Olivera-Angel

**Affiliations:** ^1^Biogenesis Research Group, Department of Agricultural Sciences, University of Antioquia, Medellín, Colombia; ^2^Colombian Institute of Tropical Medicine—CES University, Medellín, Colombia

**Keywords:** biofilm, intramammary infections, mastitis, mobile genetic elements, *Staphylococcus aureus*, virulence factors, whole genome sequencing

## Abstract

*Staphylococcus aureus* represent a serious threat to public health due to food safety, antibiotic resistance, and the potential zoonotic transmission of strains between dairy cattle and humans. Biofilm formation by *S. aureus* results in chronicity of the infections which confers protection against the immune response and antibiotics. Likewise, biofilm allows the exchange of mobile genetic material among different strains through microbial interactions inside the matrix. In Colombia, where *S. aureus* continues to be one of the main pathogens isolated from bovine intramammary infections and where milking by hand is highly frequent, there are knowledge gaps on the zoonotic potential of the strains. Therefore, the aim of this work was to characterize genotypically and phenotypically the *S. aureus* Sa1FB strain with strong biofilm production and to perform genomic and phenotypic comparisons with other relevant *S. aureus* strains (native and references strains). These results show a highly productive strain of biofilm and a low ability of cell invasion compared to the other two native strains. In addition, high genomic similarity between *S. aureus* Sa1FB and the reference strains was observed, despite of the differences reported at the clinical level. However, Sa1FB exhibited special features in terms of mobile genetic elements, highlighting its ability to accept foreign genetic material. Indeed, this could increase mutation, pathogenesis, and adaptability to new hosts, representing a risk for people in contact with the milk obtained from animals infected with these strains. These results present the relevance of surveillance for early detection of emergent clones with zoonotic potential, which reduces the risk of occupational exposure and their spread in the community.

## Introduction

*Staphylococcus aureus* has been described as a commensal pathogen from humans, and different animal species such as dairy cattle and other livestock (1, 2). It is also the most frequent agent associated with bovine mastitis worldwide (3, 4). Moreover, this Gram-positive bacterium can cause in humans a wide variety of clinical conditions including skin diseases, bacteremia-toxic syndrome, and food diseases (2, 5). On the other hand, in dairy cattle, *S. aureus* mainly produces intramammary infections (IMI) (6).

*S. aureus* represent a serious threat to public health due to food safety, antibiotic resistance, and the potential zoonotic transmission of strains between dairy cattle and humans (7). Zoonotic transfer of this pathogen between both hosts can occur by direct contact or through the food chain (8).

In Colombian specialized dairy herds, hand milking has been reported on a range between 43.6 and 77.7% (9, 10), representing a high risk for milkers in acquiring the bacterium during the milk harvest. In addition, ~41% of the raw milk obtained in the country is commercialized under informal conditions and without pasteurization, which increases the probability of spread of this pathogen among people without exposure to livestock (11).

As a versatile microorganism, *S. aureus* has become a more virulent and resistant pathogen to antimicrobials in both human and animal populations (5). This strong potential of multi-species colonization has been recognized as a product of the presence of multiple putative virulence factors (cell surface adhesins, extracellular enzymes, biofilm, cell invasion, and toxins). These virulence factors have a complex regulation network that allows the adaptation and survival in the host (7, 8). Within these virulence factors, biofilm formation is one of the main determinants, because this confers protection to both host's immune response and to antimicrobials (12). Biofilm formation results in chronicity of the infections and allows the development and transfer of antimicrobial resistance, due to microbial interactions inside the biofilm (12, 13). Another *S. aureus* factor associated with persistent infections, is the cell invasion, since this mechanism also allows it to evade the immune response and the antibiotics (14).

The whole genome sequence (WGS) analysis and the determination of the multilocus sequence typing (MLST) profile has been integrated to the genomic surveillance of potential zoonotic bacteria like *S. aureus* (15). Moreover, these methods had been used for the identification of the population structure and potential shifts in the genomic configuration (3, 8). Analyses of the MLST sequence types have revealed a highly clonal population (16) and clearly distinct clonal complexes (CCs) associated with specific hosts and environments (17–19).

The host diversity of *S. aureus* entails a direct zoonotic potential for humans, bringing the importance of its active surveillance for both public and animal hygiene (2). Therefore, a better knowledge of the adaption of *S. aureus* lineages is needed to assess the potential of host switching (15). The objective of this research was to characterize genotypically and phenotypically the *S. aureus* Sa1FB strain with strong biofilm production and to perform genomic comparisons with other relevant *S. aureus* genomes.

## Materials and Methods

### *Staphylococcus aureus* Strains

The Sa1FB strain was characterized previously genotypically and phenotypically (20). The ability to form biofilm (phenotype) on microplates and the amplification of the biofilm-associated genes, *ica* and *bap*, (genotype) were carried out according to protocols earlier published (21, 22). The genotypes and phenotypes identified were: Sa1FB Strong biofilm-producing (*ica* ^+^–*bap* ^+^), Sa2FB Weak biofilm-producing (*ica* ^+^–*bap* ^−^), and Sa3NF Non biofilm-producing (*ica* ^−^–*bap* ^−^). The three strains were isolated from bovine subclinical mastitis in Antioquia (Colombia).

### Cell Invasion Assay

The invasion assay was performed based on a previously described protocol (23). Clonal bovine mammary epithelial cells (MEC) (24) were cultured into a 24-well polystyrene culture plate (TrueLine, USA) using DMEM medium (Sigma-Aldrich, USA), supplemented with 10% fetal bovine serum (Thermo Fisher Scientific, USA), 5 μg/mL insulin (Sigma-Aldrich, USA), and 1 μg/mL hydrocortisone (Sigma-Aldrich, USA). Once the cells were confluent (1.5 – 2.5 × 10^5^ cells/well), these were independently co-cultured with each of the three native strains at a multiplicity of infection (MOI) of 10:1 and incubated for 2 h at 37°C in 5% CO_2_.

After incubation, the wells were aspirated to remove non-internalized bacteria and non-attached to the cell surface. The supernatants were cultured in Tripticase soy agar (TSA) (Oxoid, United Kingdom). Then, the MEC were washed three times with sterile PBS (VWR, USA) and treated using DMEM supplemented with 100 μg/mL gentamicin (Sigma-Aldrich, USA) in order to kill the extracellular bacteria.

The plates were incubated again for 2 h at 37°C in 5% CO_2_. Finally, the MEC were washed with sterile PBS and treated with 0.25% trypsin (AMRESCO, USA) and 0.1% EDTA (AMRESCO, USA) until cells detached, which were immediately lysed with 0.1% Triton X-100 (AMRESCO, USA). MEC lysates were diluted and plated on TSA. These plates were incubated at 37°C overnight. MEC without bacteria were used as negative control and the strains with DMEM as viability control. All the experiments were performed in triplicate and repeated two times.

### Biofilm Formation Assay *in vitro*

The biofilm production of the strains was induced following the standard protocol previously reported with some modifications (20, 21). The strains were transferred from stock culture into TSA and incubated at 37°C overnight under aerobic conditions. These colonies were suspended in sterile distilled water until a turbidity comparable to 0.5 MacFarland scale (~10^8^ CFU/mL) was reached. This suspension was diluted 1:100 in TSB supplemented with 1% glucose (Merck, USA) to reach a bacterial concentration of ~10^6^ CFU/mL. Then, 2 mL from the diluted suspension was aliquoted into 12-well polystyrene tissue culture microtiter plate (TrueLine, USA). Each well at the bottom contained a glass coverslip as a basis for biofilm formation. Experiments were performed in duplicate and repeated two times. The plates were incubated at 37°C for 24 h under static aerobic conditions. The next day, the wells were aspirated, and each well was washed three times with 2 mL sterile phosphate-buffered saline (PBS, pH 7.2). After washing, the glass coverslips were transferred to other plates and were washed again. The biofilm formation was verified using 2% crystal violet for 15 min. *S. aureus* strains V329 (*ica* and *bap* positive) and ATCC 6538 (*ica* positive and *bap* negative) were used as positive controls, whereas TSB with glucose was used as a negative control.

### Visualization of Biofilms by Electronic Microscopy

The observation of biofilm formed on microplates by the three native strains evaluated was carried out using scanning electron microscopy (SEM). Briefly, samples were fixed with a glutaraldehyde solution at 2.5% overnight and subsequently dehydrated using a different concentration of ethanol (50, 75, 95, and 100%). Finally, the samples were critical point dried, coated with gold and visualized in a JEOL-JSM 6490LV microscope (JEOL, Japan).

### DNA Extraction

The genomic DNA was extracted using a DNeasy Blood & Tissue kit (Qiagen, Germany) according to protocol for Gram-positive bacteria. Concentration and quality of DNA were measured using NanoDrop (ThermoFisher Scientific, USA). Extracted DNA was stored at −80°C until use.

### Genome Sequencing, Assembly, and Annotation

*Staphylococcus aureus* Sa1FB strain was sequenced (WGS) on the Illumina MiSeq platform. Pair reads of 300 bp in length were obtained after library preparation with the Illumina Nextera XT DNA Library preparation kit. *De novo* assembly was performed using the raw reads and the PATRIC (Pathosystems Resource Integration Center) (25) genome assembly service (revised service Dec 2019) using the SPAdes workflow (26), which integrates the trimming process of the reads using TrimGalore before assembly and Pilon (27) to correct assembly errors. A subsequent annotation was performed using Rapid Annotations using a Subsystems Technology tool kit (RASTtk) also found in PATRIC. The sequences were queried using the following tools available in PATRIC: VFDB and Victors (virulence factors) and CARD and NDARO (antibiotic resistance). To detect putative orthologs across genomes for comparing, we performed an OrthoMCL (28) cluster analysis using the default settings (*E-value* cutoff: 1e−5 and identity > 50%). The genome sequence of *S. aureus* Sa1FB was deposited in the public database PATRIC (https://www.patricbrc.org/view/Genome/1280.24396) with the genome ID: 1280.24396.

### Whole Genome Alignment

The assembled and annotated *S. aureus* Sa1FB strain was aligned with the complete bovine genomes of *S. aureus* RF122 and *S. aureus* Newbould 305 with default parameters of progressiveMauve and the average nucleotide identity (ANI) was calculated by MUMmer using JSpeciesWS (29).

## Results

### General Genome Features

**Figure 1:**
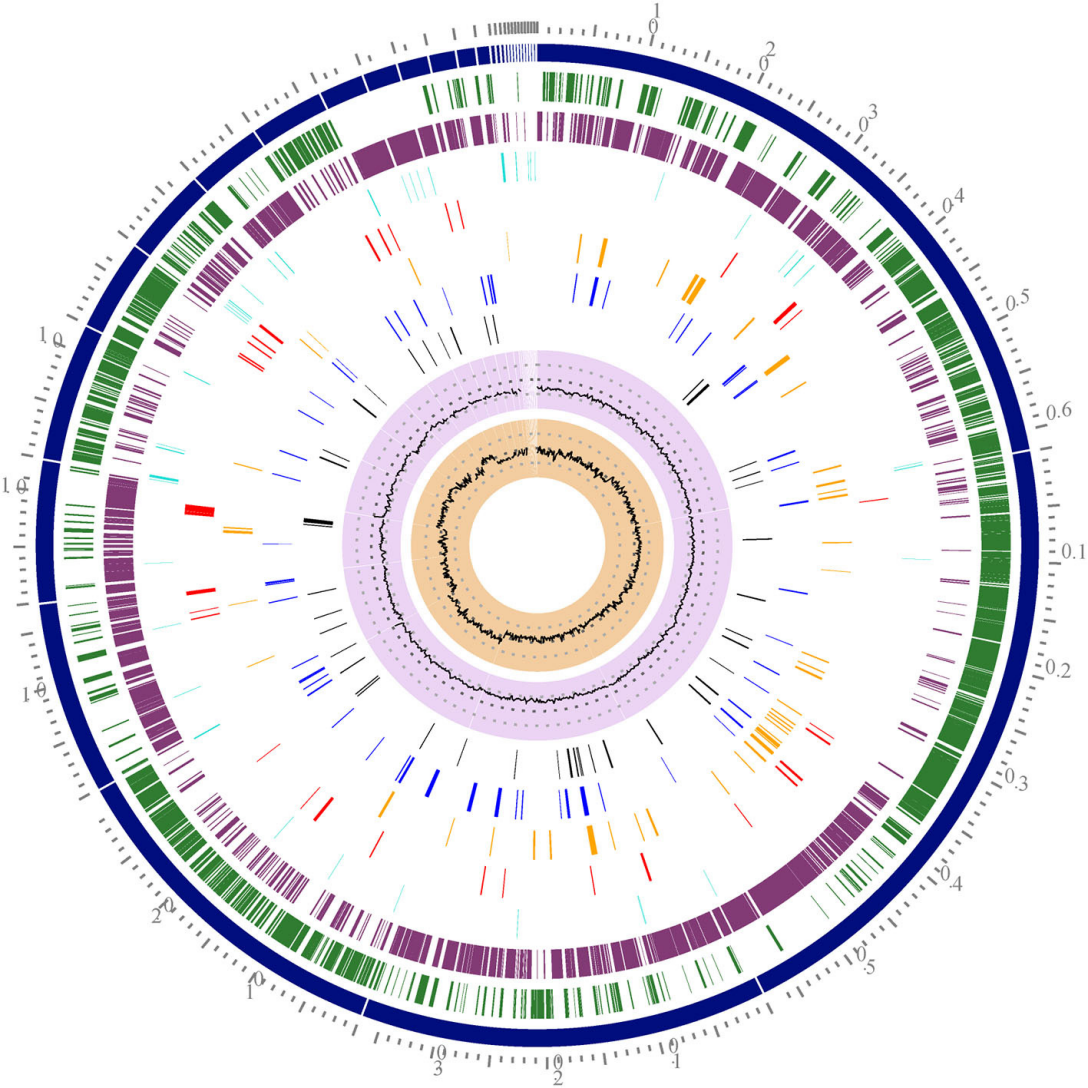
Circular representation of the *S. aureus* Sa1FB draft genome. Tracks from the outermost are as follows: Contigs, Forward CDS, reverse CDS, RNA (tRNA and rRNA), AMR genes, virulence factors, transporters, and drug targets. The two inner tracks are G+C content and GC skew. The circular map was generated by using the circular viewer of PATRIC.

Table 1 shows the *S. aureus* Sa1FB strain genome features. The sequence of the *S. aureus* Sa1FB genome draft had an estimated length of 2,745,618 bp with GC content of 32.8% and 2,632 coding sequences (CDS) as shown also in Figure 1 and Supplementary Table 1.

**Table 1:** General genome characteristics of the *S. aureus* Sa1FB strain.

**Feature**	***S. aureus* Sa1FB strain**
Genome ID (PATRIC)	1280.24396
Genome size (bp)	2,745,618
GC content (%)	32.8
Contigs	30
Total of CDS	2,632
Total of tRNA	57
Total of rRNA	9
Hypothetical proteins	507
Antibiotic resistance genes	60
Virulence factors	85
Pathogenicity island SaPIbov2	Presence
Sequence tipo (MLST)	ST126

**Figure 2:**
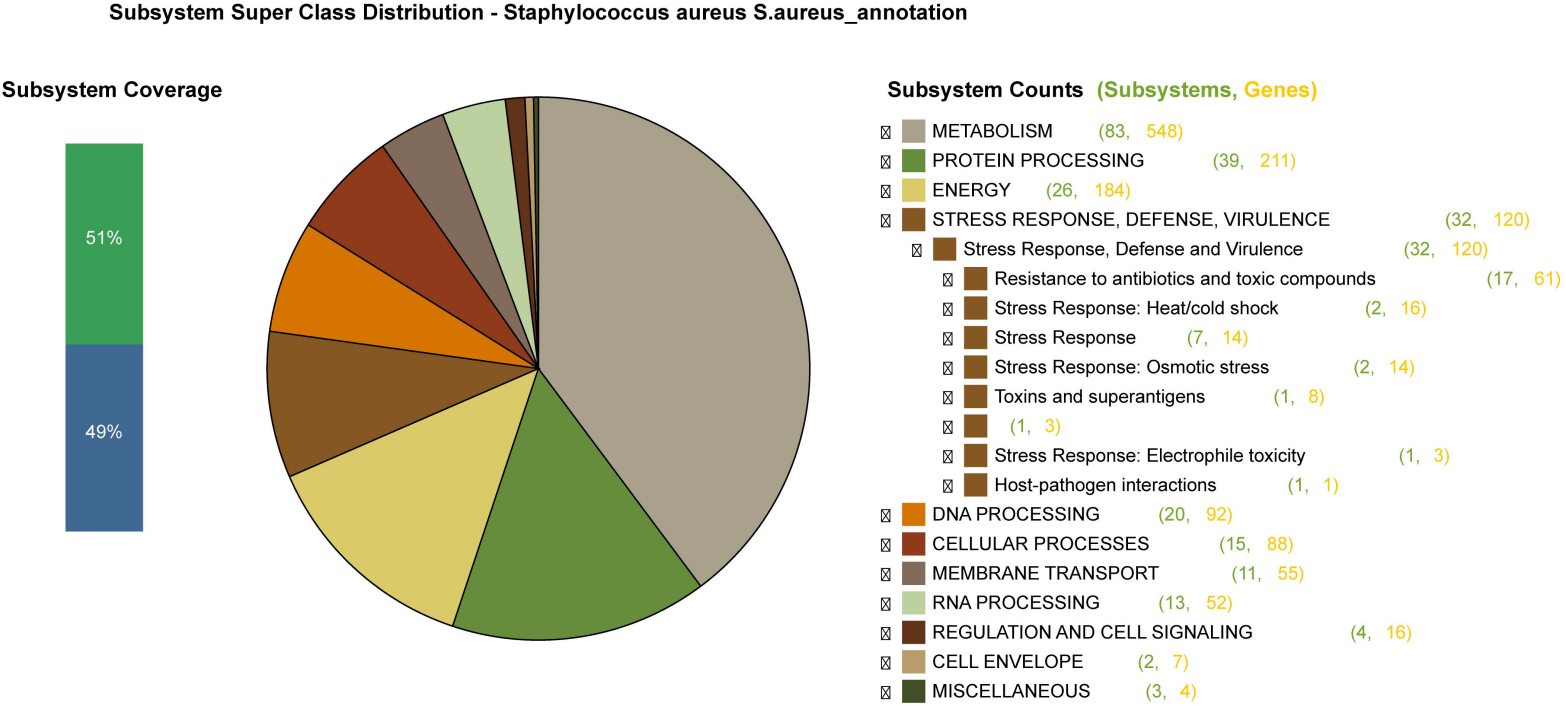
An overview of the subsystem categories of the annotated draft whole-genome of *S. aureus* Sa1FB from the RAST server. The pie chart shows the counts of genes related to each subsystem. The bar graph (on the left) determines the subsystem coverage, this is the ratio of coding sequences annotated in the SEED subsystem (51%) and outside of the SEED subsystem (49%).

Using Subsystems Technology of the RAST server it was possible to know more about the annotated genes in different biological processes and metabolical pathways (Figure 2). The predicted genes included: 548 genes involved in metabolism, 120 genes involved in stress response, defense, and virulence, and 184 genes involved in energy among other biological processes (Supplementary Table 2).

### Cluster of Orthologs Groups

**Figure 3:**
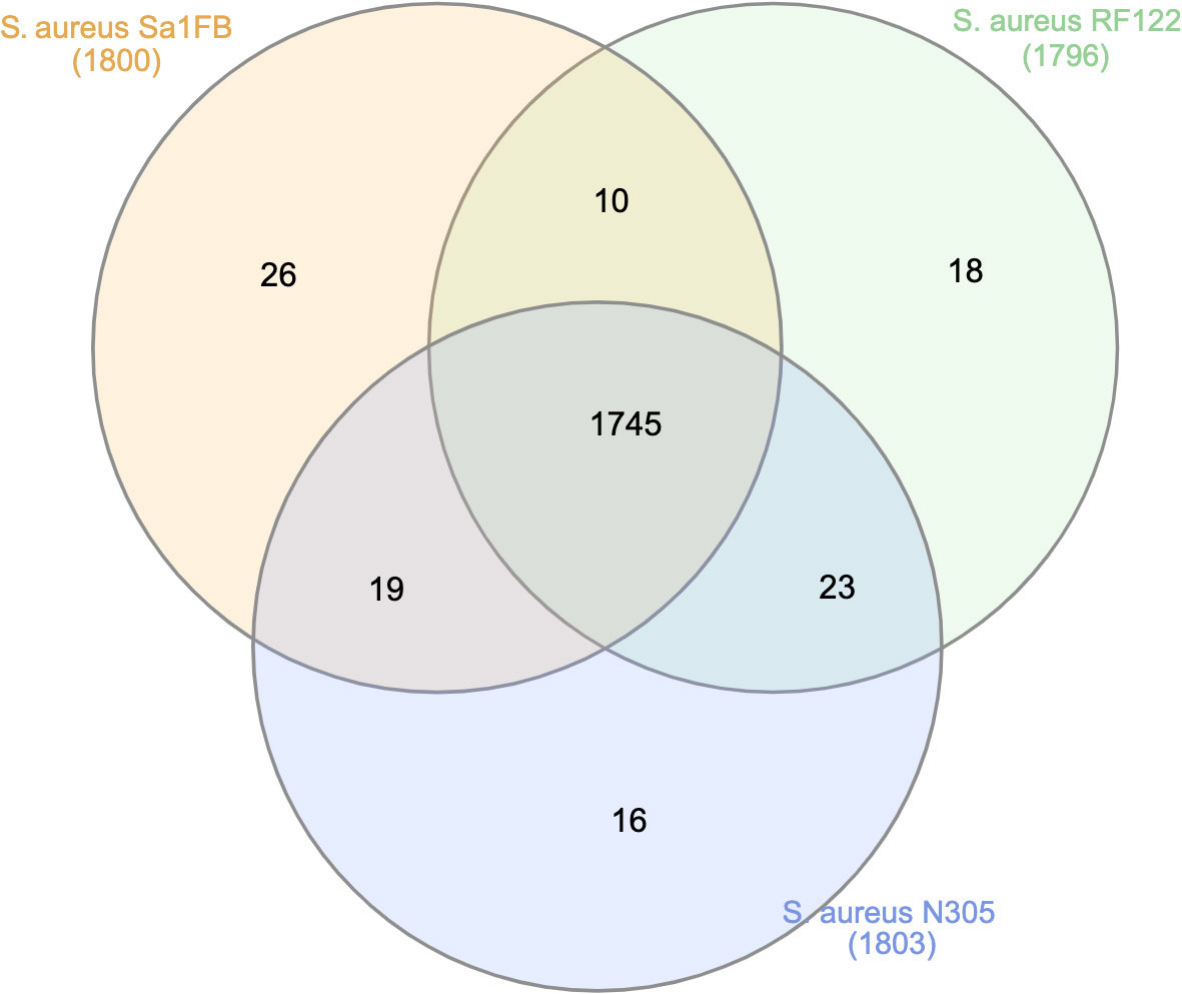
Unique and shared orthologs among *S. aureus* Sa1FB, Newbould, and RF122 strains. N305: Newbould 305.

OrthoMCL was used to arrange proteins into clusters and to identify groups of the most conserved proteins among proteomes of *S. aureus* Sa1FB, Newbould and RF122 strains. It was found that 1,745 (94%) out of the 1,857 orthologous groups were shared across three strains, showing that they probably carry similar functional capabilities (Figure 3). Twenty-six (1.4%) orthologous groups were exclusive in the *S. aureus* Sa1FB strain, which consisted of 12 protein-encoding prophage enzymes/proteins such as: helicase, terminase, endonuclease, tail tube protein, proteases, resolvase, polymerase, primase, and tail fiber proteins. Five proteins encoding for mobile element enzymes (transposases). Another finding was the identification of a hypothetical SAV0786 homolog in superantigen-encoding pathogenicity island SaPI (Supplementary Table 3).

### Whole Genome Alignment

**Figure 4:**
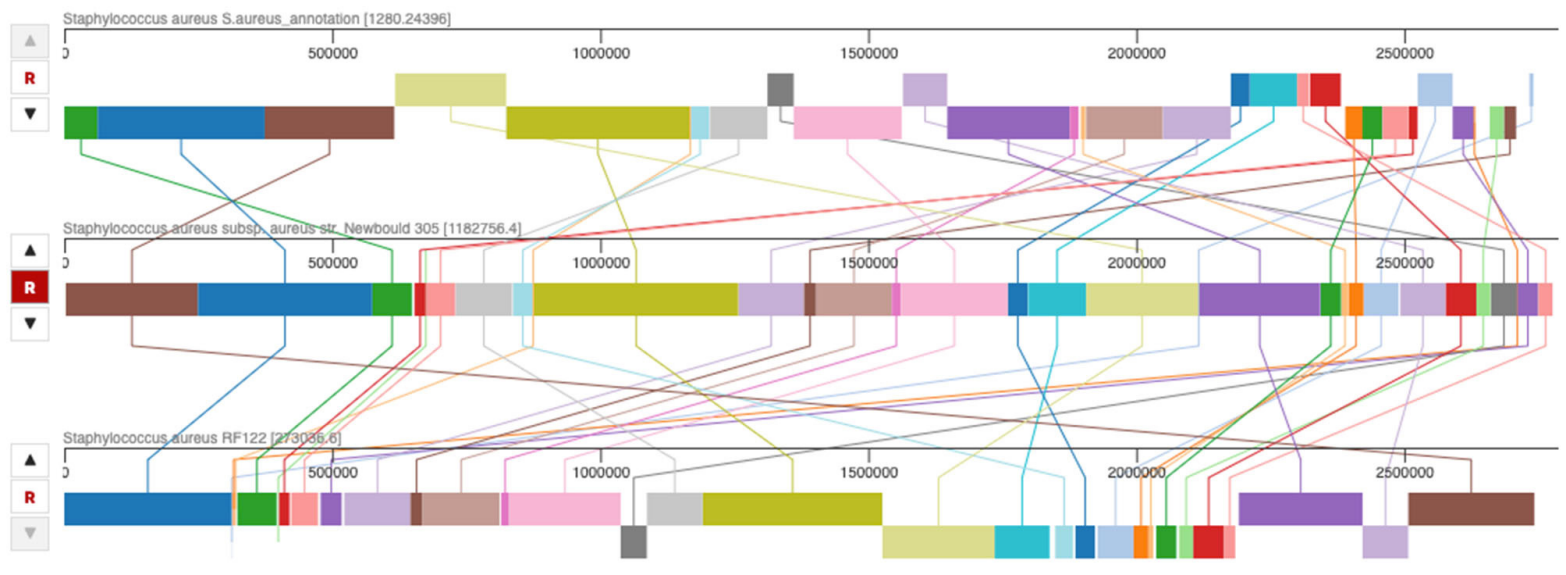
Whole genome alignment among *S. aureus* Sa1FB, *S. aureus* RF122, and *S. aureus* Newbould 305 by progressiveMauve. The lines connecting between locally collinear blocks (LCBs) indicate the blocks were conserved among three genomes.

According progressiveMauve results, we found that most of the regions of the three genomes were highly conserved (Figure 4). Each locally collinear block (LCBs) in colors was a homologous region of sequences shared across the three genomes. Despite the rearrangements across the genomes, *S. aureus* Sa1FB and *S. aureus* Newbould exhibited the highest identity (98.96%) compared with *S. aureus* RF122 (97.92%) as shown by the ANI calculation prediction based on MUMmer by JSpeciesWS.

### Biofilm Formation Genes

Subsystems Technology of the RAST identified the genes involved in the formation of biofilm in *S. aureus* Sa1FB and from these results it was possible to compare them with the biofilm formation genes predicted by the same tool available in PATRIC in the reference strains *S. aureus* Newbould and *S. aureus* RF122. The subsystems technology of RAST tool classified the biofilm formation genes in the following hierarchical classification: Superclass: Cellular processes; Class: Microbial communities; Subclass: Quorum sensing and biofilm formation; and Subsystem name: Biofilm formation in *Staphylococcus*.

For each strain (Sa1FB, Newbould, and RF122) it was possible to identify the same set of genes related with biofilm formation (Supplementary Table 4). The same 10 genes were identified across three strains and the sequence identity percentage was calculated as having *S. aureus* Sa1FB as a reference. Accordingly our annotation process was also able to identify another key gene for the biofilm formation, the gene *bap* (Biofilm associated protein) with the PATRIC identifier: fig|1280.24396.peg.2264. However, this gene was initially anotated as an hypothetical protein by the Rapid Annotations using Subsystems Technology tool kit (RASTtk) also found in PATRIC. In order to attribute function to this protein, we used a *bap* reference protein sequence deposited in the UniProt database (ID:Q79LN3) to align with our hypothetical protein, having a result of 99% of sequence identity.

Based on these results of the biofilm formation genes we could hypothesize that the *bap* gene is relevant for the biofilm formation in the Sa1FB strain, the presence of this gene being the main difference in the gene repertory related with biofilm formation across the three strains (Sa1FB, Newbould, and RF122).

### Antimicrobial Resistance

Antimicrobial resistance (AMR) phenotypes of *S. aureus* Sa1FB refer to the resistance or susceptibility to one or more antibiotics. According to the predictions on AMR phenotypes performed in PATRIC, *S. aureus* Sa1FB is susceptible to ciprofloxacin, clindamycin, erythromycin, gentamicin, methicillin, tetracycline, and trimethoprim sulfamethoxazole and is resistant to penicillin. These phenotypes were verified in the laboratory by Vitek®2 system (AST-GP79 card, bioMérieux, France), except for ciprofloxacin. In this case, two cephalosporins (cepaholotine and ceftiofur) were tested. AMR genes refer to genes implicated or associated with the resistance to one or more antibiotics. According to computational predictions based on CARD and NDARO databases *S. aureus* Sa1FB showed 60 genes likely related with resistance to different antibiotics (Supplementary Table 5).

### Virulence Factors

Eighty-five genes were identified with potential virulence factors (Supplementary Table 6). Among the different features of the virulence factors it was possible to identify genes related with the following characteristics: immune evasion, toxins, secretion system type VII, adherence, iron and heme uptake, and proteases. *S. aureus* Sa1FB showed specific virulence factors compared with the other two bovine strains, these factors were: capsular polysaccharide synthesis enzyme Cap8E and fibronectin binding protein FnbB.

### Cell Invasion

The percentage of cell invasion per each isolate ranged from 0.1 to 0.6%. Table 2 shows the percentages of cell invasion per strain.

**Table 2:** Percentage of cell invasion by strain.

**Strain**	**No. of CFU internalized**	**% of CFU internalized**
Sa1FB	3,000	0.1
Sa2FB	6,000	0.2
Sa3NF	18,000	0.6

*No, number; %, percentage; CFU, colony forming units*.

### Biofilm Morphological Characteristics by Electronic Microscopy

**Figure 5:**
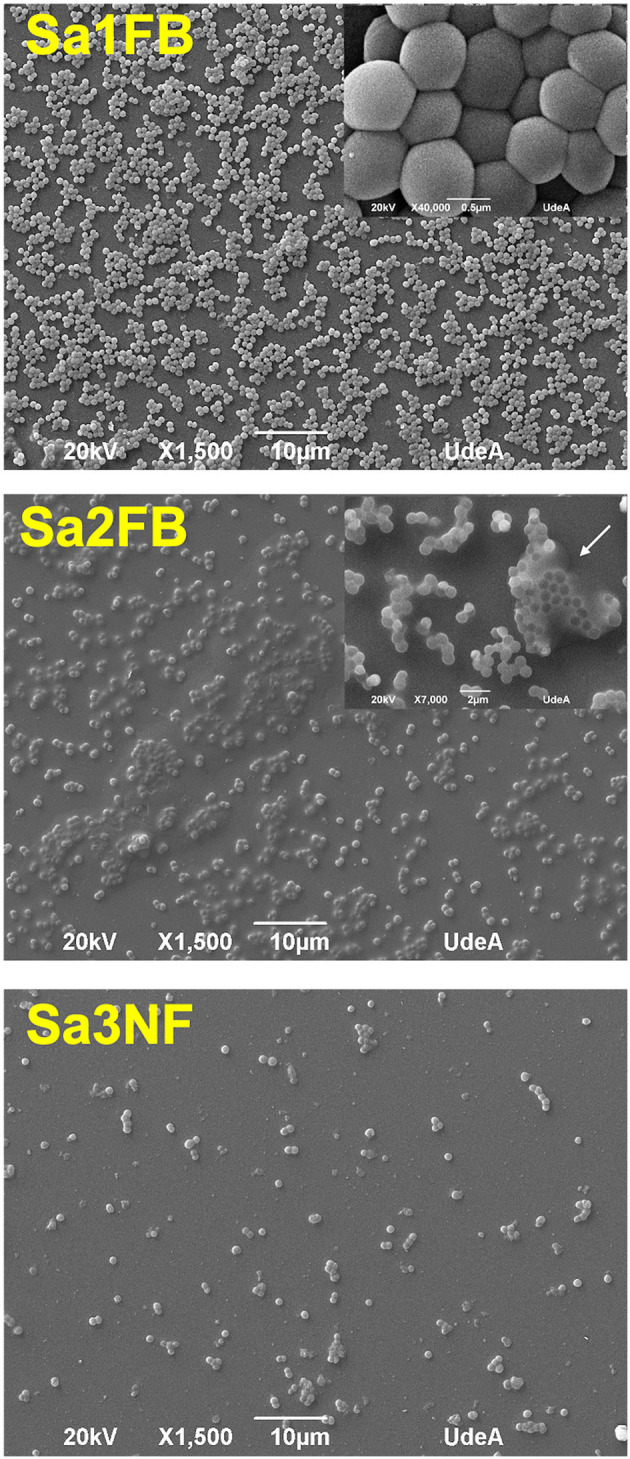
Scanning electron microscopy images of the biofilms formed by Sa1FB and Sa2FB strains. The white arrow in the Sa2FB image shows the extracellular matrix. The three images showed the differences in the ability to form biofilm: Sa1FB was strong biofilm-forming, Sa2FB was weak biofilm-forming, and Sa3NF was non biofilm-forming.

Visual inspection of the three strains by SEM, allowed us to identify considerable morphological differences (phenotypes) among these strains (Figure 5). Interestingly, the Sa1FB strain formed a biofilm in which the bacterial cells were not embedded in an extracellular matrix, despite of being a carrier of the *ica* operon. In contrast, the bacterial aggregates were very compact and free of the matrix, which is a typical feature of strains that harbor the *bap* gene.

With respect to the Sa2FB strain, the bacterial cells appear to be embedded inside an extracellular matrix, a typical feature of this genotype (Figure 5). Regarding the cellular density, less biomass was present in this isolate compared to Sa1FB, in agreement with the initial characterization of the isolates, which were weak and strong biofilm-forming, respectively.

The Sa3NF strain, corresponded to the genotype and phenotype described (*ica* ^−^–*bap* ^−^), which did not form biofilm (Figure 5).

## Discussion

The aim of this research was to explore both genotypic and phenotypic characteristics of the Sa1FB strain with strong biofilm production, and to make comparisons with other relevant *S. aureus* genomes available. These characterizations and comparisons would help to explore the zoonotic potential of this particular strain, given the virulence characteristics within the context of the milk production system in Colombia.

Alignment of the *S. aureus* Sa1FB genome with the Newbould 305 and RF122 bovine strains were recognized as causing clinical mastitis, sharing the majority of the locally collinear blocks (LCBs), indicating a substantial amount of conserved genetic information among these bovine strains. The predicted value of ANI among strains also showed a high similarity among these three strains at the genomic level. Likewise, no appreciable differences were observed among the virulence factors of the strains, agreeing with what was reported recently in a study carried out in Brazil, which compared four isolates, three of these belonging to ST126, with the same reference strains used in our study (30).

Sa1FB strain was classified as ST126, a genotype associated with infections in cows. This genotype has also been reported in the USA, Italy, and Brazil, where Brazil showed most of the reports (31–35). Furthermore, in Brazil, ST126 is one of the clones most frequently isolated from bovine mastitis and associated with persistent infections (32–34). According to PubMLST database, ST126 was assigned to CC97, since this matches at four loci with ST97 (central ST of CC97). Our results contrast with most of the reports, that place ST126 into CC126 (32–34, 36). These studies probably used other methodologies (e.g., eBURST—it is a single-linkage method) or database (www.mlst.net—currently not available) to assign the clonal complex. These discrepancies in the nomenclature could be a problem to epidemiologic surveillance of clones belonging to complexes with zoonotic potential, since CC97 is one of main *S. aureus* clonal complexes which cause bovine infections and an emerging cause of human infections. Although, the ST126 strain has only been associated with infections in cows to date, this does not guarantee that a spillover event (switch to humans) could not occur (8).

It is difficult to predict exactly when a strain will switch its genotype and adapt to a new host, because this event will depend on natural recombinations among different clones that could confer in them virulence, resistance, or immune evasion pathways (37, 38). The results obtained in this study do not predict the time it would take for the Sa1FB strain to evolve and adapt to humans, since the evidence has suggested that the recombinations can be higher *in vivo* than predicted *in vitro* or with bioinformatic tools (38). An *in vivo* study showed a high rate of transfer of mobile genetic elements (MGE) between strains isolated from animals and humans during the first 4 h of the co-colonization process (38). Also, exchange of extensive genomic regions (up to 20% of the genome) between distant lineages have been detected (37). Regarding clones of bovid origin, there are previous reports that highlight the emergence of clones that switched to humans (18, 31). One study showed the spread to humans of community-associated methicillin-resistant *S. aureus* (CA-MRSA) strains of bovine origin belonged to CC97 (39, 40). These authors detected in the strains MGE that conferred antimicrobial resistance and the capacity to evade the immune response in humans, key factors to survive and transmit among humans (40). In Denmark, cases of infected humans with strains from CC97 increased 11-fold between 2007 and 2011 (40). Likewise, other studies have also highlighted the rapid increase of the number of people colonized with *S. aureus* of the ST398 (CC398), which is a genotype shared by pigs (primary host), cattle, chicken, horses, and humans (mainly in farmer working) (41, 42). The ST398 had been recognized as a livestock-associated MRSA (LA-MRSA) strain. This clone generally is transmitted from pigs to humans, but rarely from person to person (42). However, a recent report from Denmark described a severe case of infection with LA-MRSA CC398, which was presented with bacteremia and an epidural abscess (42). Since the patient was not exposed to livestock, the authors suggested that the transmission was from person to person (42). These findings demonstrate the high ability of adaptation in *S. aureus* and its pathogenic potential in different hosts. Also, it provides strong evidence that livestock could act as a potential source of new human-pathogenic *S. aureus* strains, like other clones belonging to CC97.

The genotypic and phenotypic analysis performed on our strain (Sa1FB) showed different virulence factors; among these, the high ability to form biofilm. This mechanism allows resistance to antibiotics and disinfectants, as well as the evasion of the immune response of the host. These characteristics allow this pathogen to persist for a long time in both biotic and abiotic environments (12). In infections caused by biofilm-forming *S. aureus* strains, the concentration of antibiotic required to kill them can be 10-fold higher compared to planktonic bacteria (43–45). In addition, exposure to antibiotic selection pressure in biofilms has also been linked to the development of antibiotic resistance in this pathogen (46). According to the phenotypic predictions performed in PATRIC and confirmed by Vitek®2 system, *S. aureus* Sa1FB is susceptible to different antibiotics and resistant to penicillin. Previous works performed in the region showed the same behavior (penicillin resistance) in the evaluated *S. aureus* strains (47, 48). Despite low antibiotic resistance found in Sa1FB, a study reported that *S. aureus* strains carrying the *bap* gene were less susceptible to antibiotic treatments when forming biofilm *in vitro* and more persistent in the bovine mammary gland (22).

We found that the Sa1FB strain was a carrier of *ica, fnbB*, and *bap* genes, which had been widely associated with the biofilm formation process (13, 22, 49, 50). Both *fnbB* and *bap* were only identified in Sa1FB but not in the reference strains included. This result is in agreement with what was identified in a study which compared eight *bap* positive strains with the reference strain V329. The authors also detected these genes in the isolates evaluated (35). The *bap* gene encodes the biofilm-associated protein (Bap), involved in intercellular adhesion and, subsequently, in bacterial accumulation. This gene generally confers a strong biofilm-forming phenotype and is located in a transposon inserted in the SaPIbov2 mobile pathogenicity island (22, 51). This gene was the main difference in the gene repertory related with biofilm formation across three strains (Sa1FB, Newbould and RF122). In contrast to what is commonly reported, *bap* positive strains are usually isolated from bovine mastitis, a research carried out in Italy found this genotype in pigs, suggesting that they potentially have the ability of moving across different hosts (39). It has also been demonstrated in experimental infections that *bap* positive strains cause more persistent infections in bovines and present higher resistance to antibiotics in comparison to strains that did not harbor this locus (22). Recently, we highlighted the virulence potential of this genotype, since we reported that strains carried the *bap* gene were more capable of producing strong biofilms than *bap*-negatives (20).

Regarding *ica* operon, which encodes the polysaccharide intercellular adhesion (PIA) factor, which is the most common mechanism used by *S. aureus* for biofilm formation, since most of the clinical isolates involved in both human and animals infections carry this locus (13). Despite the Sa1FB strain also harboring the *ica* operon, the phenotype observed by electronic microcopy did not show the extracelullar matrix characteristic of PIA-dependent biofilm. In contrast, we observed a very compact bacterial aggregate where this matrix is not evident, suggesting that in the presence of both loci, *bap* gene drives the biofilm formation. These findings are consistent with other reports, who have described two biofilm phenotypes, one where cells are embedded by an extracellular matrix (PIA-dependent) and the other based on proteins such as fibronectin-binding proteins (FnBPs) and Bap (PIA-independent) (13, 49). A research also concluded that the *bap* gene was sufficient to generate biofilm, even in the absence of the *ica* operon (22). It is important to highlight that SEM does not determine the presence of PIA. For it is necessary to use other techniques such as antibodies PIA-specific, staining or quantification of sugar content in the matrix.

On the other hand, the *fnbB* gene encodes for fibronectin-binding protein B (FnBPB), a molecule that plays an important role in PIA-independent biofilms (13, 49, 52). This protein is involved in the primary attachment phase to fibronectin, elastin, and fibrinogen, as well as in the accumulation process in biofilm formation (13, 52). It has also been demonstrated that FnBPB favors cell invasion and protects against the antimicrobial activity of histones released during the neutrophils extracellular traps (NETs) (23, 53). Since we did not evaluate the presence of this marker in the other two native strains tested, we cannot discuss its influence in the invasion capacity. A study in which a mutant strain for *fnb* was generated showed significantly impaired colonization and biofilm formation in the animal model used (54). Some studies have concluded that genes that promote better colonization of *S. aureus* and better immune response evasion can help its spread from person to person (37, 55).

Biofilms have been recognized as niches where gene transfer among bacteria can occur, conferring them antimicrobial resistance and the capacity for adaptation to different hosts and environments (56–58). Several studies have revealed that horizontal gene transfer and biofilms are related processes and have high relevance in bacterial adaptation and evolution (56, 57). The acquisition of the great majority of MGE is performed through horizontal gene transfer, including the *S. aureus* pathogenicity islands (SaPIs), that can be disseminated in bacterial populations through phages (59). We exclusively found in Sa1FB material associated with MGE (prophages and transposases), confirming its ability to accept genetic material. MGE transfer is usually higher within biofilms than in planktonic cells, since biofilms provide optimal conditions for bacterial interactions, due to the close proximity of cells within this structure and because the matrix can accumulate different chemical compounds that facilitate the process (e.g., communication signals and extracellular DNA) (60). Hence, biofilms have a high impact on the dissemination of MGE in bacteria of clinical and industrial relevance, such as *S. aureus*. In a study performed in the United Kingdom demonstrated that biofilm formed by this specie significantly increased horizontal transfer of plasmid-borne antibiotic resistance (58). Other studies reported that staphylococci within biofilms increased mutability, which could also accelerate the spread of resistance and adaptability traits among strains that coexist in these biofilms matrices (61). Similarly, other authors detected some horizontal transfer and antibiotic resistance genes in the staphylococcal clinical strains collected from biofilms (57). According to published results in Chile, biofilms produced on surfaces from milking equipment can act as source of *S. aureus* contamination for bulk tank milk and cows. This same study found that several genotypes can inhabit inside the same biofilm simultaneously (62). Due to the high ability to form biofilm in our strain, it can generate this structure on surfaces in contact with milk during milking process or cooling, where it could interact and MGE exchange with zoonotic strains. Recently, we conducted a study in Antioquia (Colombia), in which 97 strains isolated from IMI were genotyped by spa typing. The results showed that 50% of bovines were infected with genotypes that also caused infections in humans, a fact that demonstrated close proximity between human and bovine strains in the region, as well as the high risk of a spillover event (63).

Cell invasion is another way in which *S. aureus* can avoid antibiotics and the immune response in hosts (14). In our study, the Sa1FB strain showed the lowest invasion percentage, supporting results previously informed by other authors, who evidenced that Bap promoted the adhesion and biofilm formation but prevented cellular internalization (64). We observed that as the ability to form biofilm in the strains tested decreased, the percentage of cell invasion increased, indicating that the adhesion and formation of bacterial accumulations on epithelial cells probably reduced the entry of *S. aureus* into them. Similar results were observed in a study conducted in Brazil, since they found that the strain with the largest production of biofilm presented the lowest invasion rate; whereas, the strain that showed poor biofilm formation had the highest invasion rate. However, they did not observe any relation between the invasion capacity and *bap* gene presence (65). Contrary to our finding, there are reports that did not find an association between capacity to produce biofilm and the invasiveness (23, 66).

In Colombia, where this pathogen has been reported as one of the main causes of IMI, the importance of cows as a source of zoonotic *S. aureus* strains is unclear. The risk of transmission of this pathogen to people working in close contact with livestock, especially for those who perform milking by hand, is probably high. These facts highlight the relevance of surveillance for early detection of emergent clones and the application of biosecurity actions in the agricultural setting that reduces the risk of occupational exposure and their spread in the community (40).

## Conclusions

This study shows the high genetic similarity between *S. aureus* Sa1FB and the reference strains, despite the differences reported at the clinical level. Nevertheless, the Sa1FB strain exhibited special features in terms of MGEs, highlighting its ability to accept foreign genetic material. Indeed, this could increase its mutability, pathogenesis, and adaptability to new hosts, which represents a risk for milkers and people in close contact with the milk obtained from animals infected with these strains. Furthermore, the high ability of Sa1FB to form biofilm would generate the proper environment where the exchange of genetic material among strains could occur.

These findings highlight the relevance of surveillance for the detection of emergent clones with zoonotic potential, which reduces the risk of occupational exposure and their spreading in the community.

## Data Availability Statement

The datasets generated in this study are deposited in PATRIC (patricbrc.org) under the genome ID: 1280.24396 (https://www.patricbrc.org/view/Genome/1280.24396).

## Author Contributions

GT and MO-A contributed to the conception, design of the study, and were responsible for funding acquisition. GT and KV carried out the experiments. GT, YC-A, JR-V, and MO-A carried out the analyses data. All authors wrote, contributed to manuscript revision, and approved the submitted version.

## Conflict of Interest

The authors declare that the research was conducted in the absence of any commercial or financial relationships that could be construed as a potential conflict of interest.
