# Supplementary material for: Phenotypic Characterization and Whole Genome Analysis of a Strong Biofilm-Forming Staphylococcus aureus Strain Associated With Subclinical Bovine Mastitis in Colombia
Source: Front Vet Sci. 2020 Sep 4;7:530. doi: 10.3389/fvets.2020.00530 (PMC7500091; doi:10.3389/fvets.2020.00530)
Supplement: Supplementary file 4 [file Table_4.DOCX]

**Supplementary table 4**. Biofilm formation genes

| Biofilm formation genes | Product | PATRIC IDs (per strain) | | | Sequence identity (%) | |
| --- | --- | --- | --- | --- | --- | --- |
|  | | Sa1FB | Newbould | RF122 | Newbould | RF122 |
| icaA | Polysaccharide intercellular adhesin (PIA) biosynthesis N-glycosyltransferase IcaA (EC 2.4.-.-) | fig\|1280.24396.peg.379 | fig\|1182756.4.peg.541 | fig\|273036.6.peg.2679 | 100 | 99 |
| icaB | Polysaccharide intercellular adhesin (PIA) biosynthesis deacetylase IcaB (EC 3.-.-.-) | fig\|1280.24396.peg.377 | fig\|1182756.4.peg.543 | fig\|273036.6.peg.2681 | 100 | 98 |
| icaC | Polysaccharide intercellular adhesin (PIA) biosynthesis protein IcaC | fig\|1280.24396.peg.376 | fig\|1182756.4.peg.544 | fig\|273036.6.peg.2682 | 99 | 99 |
| icaD | Polysaccharide intercellular adhesin (PIA) biosynthesis protein IcaD | fig\|1280.24396.peg.378 | fig\|1182756.4.peg.542 | fig\|273036.6.peg.2680 | 100 | 92 |
| icaR | Biofilm operon icaABCD HTH-type negative transcriptional regulator IcaR | fig\|1280.24396.peg.380 | fig\|1182756.4.peg.540 | fig\|273036.6.peg.2678 | 99 | 99 |
| tcaR | Teicoplanin-resistance associated HTH-type transcriptional regulator TcaR | fig\|1280.24396.peg.1596 | fig\|1182756.4.peg.1732 | fig\|273036.6.peg.2356 | 82 | 82 |
| sarA | Staphylococcal accessory regulator A (SarA) | fig\|1280.24396.peg.1967 | fig\|1182756.4.peg.1148 | fig\|273036.6.peg.617 | 100 | 100 |
| sigB | RNA polymerase sigma factor SigB | fig\|1280.24396.peg.2538 | fig\|1182756.4.peg.2739 | fig\|273036.6.peg.2055 | 99 | 99 |
| atl | N-acetylmuramoyl-L-alanine amidase (EC 3.5.1.28) / Endo-beta-N-acetylglucosaminidase (EC 3.2.1.96) @ Bifunctional autolysin Atl | fig\|1280.24396.peg.1305 | fig\|1182756.4.peg.1501 | fig\|273036.6.peg.980 | 99 | 98 |
| SAOUHSC_00673 | Transcriptional regulator of biofilm formation (AraC/XylS family) | fig\|1280.24396.peg.1941 | fig\|1182756.4.peg.1197 | fig\|273036.6.peg.665 | 99 | 98 |
